# From the Eye of the Storm to Epidemiological Footprints After the Floods: Viral, Vector-Borne, and One Health Risks Post-Hurricane Melissa in Jamaica

**DOI:** 10.3390/v18060605

**Published:** 2026-05-26

**Authors:** Kirk O. Douglas, Gail Ranglin-Edwards

**Affiliations:** 1 Centre for Biosecurity Studies, The University of the West Indies, Cave Hill, St. Michael BB11000, Barbados; 2 Health Service Corps, Jamaica Defence Force, Up Park Camp, Kingston 5, Jamaica; granglinedwards@hsph.harvard.edu

**Keywords:** hydrometeorological disasters, hurricanes, One Health, Jamaica, Caribbean, zoonoses, wildlife, systems thinking, arboviruses, leptospirosis, hantavirus

## Abstract

Hurricanes cause severe impacts on lives, livelihoods, and essential systems. Hurricane Melissa impacted Jamaica as a Category 5 cyclone, resulting in estimated losses of approximately 41% of national GDP (US$8.8 billion) and eliciting widespread damage to housing, healthcare, agriculture, and urban infrastructure. Agriculture sustained heavy losses, with 41,000 hectares of damaged farmland and the loss of more than 1 million livestock animals. These impacts resulted in exposed animal closures with biological hazards. Using systems thinking, the PESTHEEL framework, and a One Health lens, we argue for viewing Hurricane Melissa as series of cascading inter-related One Health threats of waterborne and vector-borne diseases, zoonoses, antimicrobial resistance, degraded indoor and outdoor air quality, chemical pollution, and shifting migration and border dynamics. These each unfold at different timings. A structured synthesis for Jamaica and other Caribbean Small Island Developing States is provided by integrating systems thinking, One Health, and the PESTHEEL framework. Immediate and lagged risk pathways are identified, and practical risk reduction actions are proposed to support anticipatory, multisectoral recovery: enhanced syndromic, laboratory, wastewater, vector, and rodent surveillance; resilient WASH and shelter systems; non-insecticidal and integrated vector management; biosecure aid and border protocols; environmental toxicology monitoring; and climate–health intelligence.

## 1. Introduction

Hurricanes are devastating natural disasters that exact a heavy toll on lives and livelihoods by destroying property and infrastructure. The intensity and frequency of climate-driven hydrometeorological events like hurricanes and cyclones continue to increase globally [1,2]. Caribbean island territories, much like other Small Island Developing States (SIDSs), are forecasted to experience multiple and compound climate-related risks [1]. Studies have revealed an increased likelihood of higher temperatures, more intense rainfall activity, and more frequent and intensive weather systems [3].

Hurricane Melissa struck Jamaica on 28 October 2025 as a powerful Category 5 cyclone, with maximum sustained winds reported at 185 mph (295 km/h), 15–30 inches of rainfall and storm surges up to 13 feet in the worst affected areas [4]. The storm exposed the full national population of approximately 2.8 million people; directly affected more than 1.5 million people; damaged hospitals, water systems, roads, telecommunications and power infrastructure; and produced estimated physical damages of US$8.8 billion, equivalent to 41% of Jamaica’s 2024 GDP [5]. These figures support the premise that Hurricane Melissa was not only an extreme meteorological event but a national systems shock, with direct implications for infectious disease risk, environmental health, food security, health service continuity, and biosecurity governance.

There is an increasing concern among Caribbean SIDS that freshwater shortages combined with more droughts, hurricanes, and tropical storms could result in a decline in sanitation and hygiene standards [6]. Under such circumstances, there is an increased risk of exposure to multiple health threats, specifically communicable diseases. With increasing climate variability, the expectation is a concomitant increase in the frequency and intensity of infectious disease outbreaks and epidemics, including dengue, leptospirosis, Zika, hantavirus, Chikungunya, and Mayaro and Oropouche fevers [7,8,9,10,11]. Economic evaluations and impact assessments are necessary to comprehend the ramifications of climate-sensitive infectious diseases in the Caribbean [12].

Hurricane Melissa is an exemplar of a multifaceted One Health stress scenario where human, animal, plant, and environmental health risks are intricately interlinked over time, not only in the short term but also over the long term, with acute and chronic impacts [13,14,15]. We present a synthesis of integrated post-flood infectious diseases, arboviral and rodent-borne risks, mould and air-quality hazards, chemical risks, agricultural zoonoses, AMR, and aid-related biosecurity in a single analytical framework for Caribbean SIDS. Immediate institutionalisation of systemic One Health-oriented governance that encompasses surveillance and non-insecticidal vector control, climate–health intelligence, biosecure aid protocols, resilient WASH and housing, and equity-focused ethics is strongly recommended to effectively manage the health security complexities of Hurricane Melissa and future climate-amplified hydrometeorological events.

## 2. Methods and Analytical Approach

We have used “One Health” to refer to the integrated analysis of human, animal, plant, and environmental health. A systems thinking approach was adopted to examine feedback loops and time lags, reinforcing pathways and cross-sector dependencies rather than treating hazards as independent linear events [16,17]. We used PESTHEEL as a structured analytic framework covering political, economic, social, technological, health, environmental, ethical, and legal dimensions of risk [18]. The PESTHEEL assessment was developed by mapping each post-hurricane risk pathway to the sectoral drivers, governance gaps, and actionable interventions most relevant to Jamaica’s disaster response and recovery [18]. Tables and figures were generated by the authors through a purposive narrative synthesis of (i) peer-reviewed evidence on post-hurricane infectious disease, vector ecology, rodent ecology, AMR, toxicology, and air quality; (ii) publicly available Jamaica-specific situation reports, advisories, and damage estimates; and (iii) the authors’ One Health and biosecurity expertise. Where Jamaica-specific quantitative data were unavailable due to data poverty or inaccessibility, this limitation is explicitly stated, and comparative evidence from analogous hurricane or flood settings is used cautiously as a proxy rather than as direct proof.

## 3. Results

### 3.1. Water, Sanitation, Hygiene, and Gastrointestinal Disease Risks

Acute gastroenteritis is a global health disease, with an estimated 700 million annual cases occurring in children (less than 5 years of age), causing limited deaths in developed countries but over 2 million deaths in developing countries [19,20]. A diverse group of pathogens are aetiological agents of acute gastroenteritis, including enteric viruses, bacteria, and parasites, which cause symptoms of nausea, vomiting, abdominal pain, fever, and acute diarrhoea [19,20]. Flooding related to Hurricanes Matthew and Florence in North Carolina, USA, during 2016 and 2018, respectively, resulted in increased acute gastrointestinal illness emergency department (AGI ED) visit rates as high as 85% and predominately affected Black and Indian minority populations [21].

The conditions in the aftermath of hydrometeorological events often facilitate the occurrence of explosive acute gastroenteritis outbreaks in vulnerable populations, such as homeless and internally displaced persons (IDPs), children under 5 years of age, and the elderly, due to damaged water, sanitation, and hygiene (WASH) infrastructure, reduced access to clean water, exposure to crowded hurricane shelters, and increased contact with compromised stormwaters carrying waste from compromised ecosystems [13,21,22]. A rapid acute norovirus gastroenteritis outbreak occurred among hurricane shelter evacuees in the direct aftermath of Hurricane Katrina in Houston, Texas, USA, where >1000 patients were treated for gastroenteritis within a space of 9 days [23]. Overcrowded conditions in hurricane shelters present serious public health challenges in effectively managing such rapid outbreaks, underscoring the necessity for rapid, sensitive, and high-quality laboratory assays to detect enteric pathogens early to aid in the reduction in disease transmission. Notably, some enteric pathogens are zoonotic and transmissible to farm animals including pigs and cows, causing disease and mortality [24,25,26,27] and further complicating public health efforts for infectious disease containment.

The Caribbean has long recognised leptospirosis as a classic post-flood disease [28,29,30], and recent outbreaks elsewhere in the region after hurricanes have been linked to floodwater exposure, with a marked rise in cases citing contact with contaminated water [31,32,33]. Hurricane Melissa’s strongest immediate impact on health in Jamaica was via water. Intense rainfall, storm surges, and flash flooding damaged or disrupted potable water systems and created new freshwater springs—especially in Western and Southern Jamaica—prompting the Ministry of Health and Wellness (MoHW) to warn citizens to treat or boil all drinking water and to avoid potentially contaminated sources [34]. The effectiveness of boil water advisories (BWAs) strongly depends on implicit public understanding and compliance, as factors such as timeliness of receipt, content of the advisory, and number of sources reporting the advisory can have a significant impact [35,36,37]. Key recommendations to increase BWA effectiveness and compliance include improvement in the tone and content of BWA notices and the use of a standard protocol to limit recall bias and accurately capture the public response [35,36,37].

Moribund animals and human remains that decompose in flooded areas pose serious contamination threats to stagnant water and exposure risks to humans [38]. Hurricane Melissa temporarily rewrote the ecology of the island of Jamaica: rivers changed course, mountainsides slid, sewage mixed with floodwater, groundwater levels rose, livestock and pets died, and millions of tonnes of debris accumulated in drains, gullies, and streets [39]. Early assessments indicated damage equivalent to nearly a third of Jamaica’s GDP, with severe impacts to five major hospitals and over 100 health centres [40]. In places like the southern parish of Manchester, Jamaica, officials warned residents to stay out of floodwaters due to sewage contamination and the risk of waterborne and vector-borne diseases, including leptospirosis [39,41]. These climate change impacts have extenuated ripple effects which include certain vector-, food-, and water-borne diseases like dengue, Chikungunya, norovirus, enterovirus, hantavirus, and leptospirosis [42].

Early post-Melissa surveillance data provide Jamaica-specific evidence that this pathway was not merely theoretical. PAHO reported that a leptospirosis outbreak was declared on 21 November 2025 [43]; by 26 November 2025, Jamaica had reported 39 confirmed, probable, or suspected leptospirosis cases after Hurricane Melissa, including 18 confirmed cases and 10 deaths, of which six were confirmed [43]. Laboratory reporting also identified 21 leptospirosis-positive cases by 26 November 2025, including 18 post-Melissa and three pre-Melissa positives [43]. These figures demonstrate a measurable increase in post-flood zoonotic morbidity, while also showing the importance of distinguishing suspected, probable, confirmed, and fatal cases in outbreak interpretation.

Floodwater in a post-hurricane Caribbean setting, with high temperatures and humidity, is far more sinister than rainwater. Floodwater contains a mixture of overflowing latrine and septic tank wastewater from damaged sewerage infrastructure, animal and human faecal matter, decomposing animal carcasses, petrochemicals and hazardous chemicals, heavy metals, and decomposing organic matter. In this toxic milieu, a diverse array of pathogens can proliferate, including *Leptospira* spp., enteric viruses, and a spectrum enteric gut microflora of *Salmonella*, *Shigella*, *Yersinia*, *Enterobacter*, *Campylobacter*, *Clostridium perfringens*, *Vibrio cholerae*, and pathogenic *E. coli* (Table 1).

Flooding in North Carolina after Hurricane Floyd in 1999 resulted in environmental contamination with faecal wastes from municipal wastewater and livestock operations [44]. This resulted in increased health risks to humans due to consumption of crops grown in faecally contaminated soil and ingestion of contaminated water. Specifically, flooded soil (post-Hurricane Floyd) demonstrated significantly higher levels of *Cl. perfringens* spores (*p* < 0.001) compared to pre-flood soil [44]. Thus, caution should be exercised in crop production in previously flooded soils post-flood events where human and animal faecal contamination may be present.

**Figure 1:**
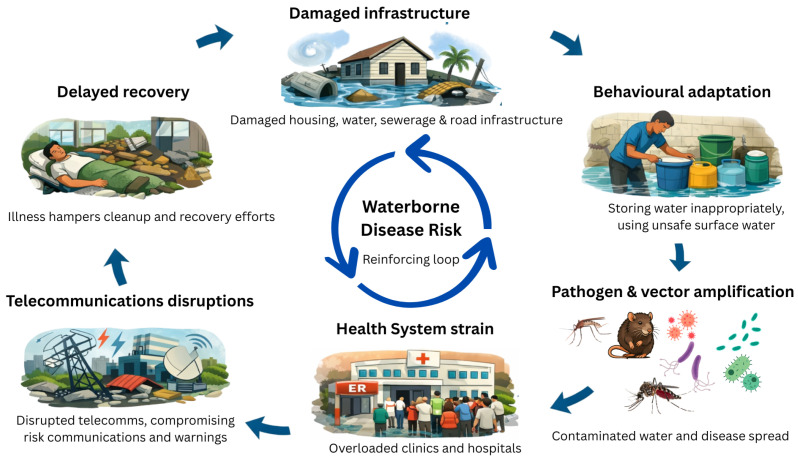
Waterborne disease risk as a reinforcing loop in the aftermath of a hurricane or hydrometeorological event. Directional arrows denote hypothesised causal pathways linking damaged housing, water, sewerage and road infrastructure to behavioural adaptations, pathogen and vector amplification, health-system strain, disrupted telecommunications and delayed recovery. The central circular arrows represent the self-reinforcing structure of the system, in which illness and impaired communication slow clean-up, risk communication and infrastructure restoration, thereby sustaining exposure to contaminated water and amplifying disease risk. Colours are illustrative only and do not denote quantitative risk levels.

Using a systems-thinking perspective, waterborne disease risks can be understood as a reinforcing loop: (1) infrastructure shock as hurricanes damage disrupts water, road access systems, and waste infrastructure, including sanitation and drainage; (2) behavioural adaptation as households aim to store water in improvised containers, collect surface waters, and use unsafe well water; (3) pathogen amplification as contaminated water, warm, tropical conditions, and poor hygiene conditions fuel diarrhoeal diseases and leptospirosis; (4) health system strain where clinics and hospitals—damaged and working below capacity—become overwhelmed with acute cases, reducing their capacity to restore routine services or carry out community outreach; (5) disrupted digital and radio communication systems in impacted areas significantly affected risk communication strategies; and (6) delayed recovery, where ill health among adults responsible for livelihood and clean-up slows debris removal and sanitation improvements, further exacerbating exposure risks. (Figure 1).

**Table 1:** A list of One Health biothreats in a post-Hurricane Melissa scenario in Jamaica.

Biothreats	Human	Animal	Plant
Viral (vertebrate)	H5N1 avian influenza or ‘bird flu’ [45]	H5N1 avian influenza or ‘bird flu’ [45,46]	
	Swine influenza (H1N1, H3N2) [47]	Newcastle disease virus (NDV) or paramyxovirus [46]	
		Porcine reproductive and respiratory virus (PRRSV) [47]	
		Swine influenza [47]	
		Aujeszky’s disease [48]	
		Parvovirus	
Viral (waterborne)	Enterovirus, norovirus, and rotavirus [49]		
Viral (rodent-borne)	Hantavirus [9]	Hantavirus [9]	
	Arenavirus [9]		
Viral (vaccine preventable)	Measles [50]		
	Influenza [51]		
	COVID-19 [52]		
Viral (Vector-borne)	Dengue virus [53]	African swine fever [54]	Cucurbit yellow stunting disorder virus (CYSDV) [55]
	Mayaro virus [56] and Zika virus [57]	Bluetongue virus [58]	Cacao yellow vein-banding badnavirus [55]
	Yellow fever virus [59]	Classic swine fever [48]	Tomato yellow leaf curl begomovirus [55]
	Chikungunya virus [60]	West Nile virus [61]	Sweet potato feathery mottle potyvirus [55]
	Oropouche virus and Melao virus [11]		Cacao mild mosaic badnavirus [55]
	West Nile virus [62]		Cucurbit yellow stunting disorder virus (CYSDV) [55]
			Citrus tristeza virus (CTV) [55]
			Sweet potato chlorotic stunt closterovirus [55]
			Papaya ringspot potyvirus [55]
Bacterial (vector-borne)	*Leptospira* spp. [32]	*Ehrlichia ruminantium* (heartwater) [63]	Citrus huanglongbing (HLB) [64]
	*Mycobacterium tuberculosis* (TB) [65]	Enteric bacteria (*Salmonella*, *Shigella, E. coli, Campylobacter*, etc.)	
	Enteric bacteria (*Salmonella*, *Shigella*, *E. coli*, *Campylobacter*, etc.)		
	*Clostridium perfringens* [44]		
Bacterial (water-borne)	*Vibrio cholerae* (cholera) [66]		
Bacterial (wound)	*Vibrio vulnificus* [67]		
	*Clostridium tetanae* [68]		
	*Streptococcus pyogenes* [69]		
Bacterial (vaccine preventable)	*Corynebacterium diphtheriae* (diphtheria) [70]		
Fungal	*Aspergillus niger*, *Penicillium* spp., *Trichoderma*, and *Paecilomyces* [71]		*Fusarium* wilt Tropical Race 4 (TR4) [72]
	*Histoplasma* [73]		
	*Candida auris* [74]		
Parasite	Malaria [75]		
	*Angiostrongylus cantonensis* (eosinophilic meningitis) [76]		

**N.B.** Table 1 was constructed as a risk-mapping table rather than a prevalence table. Pathogens and biothreats were included when there was (i) evidence of occurrence in Jamaica, the Caribbean, or ecologically comparable tropical settings; (ii) plausible amplification after flooding, displacement, debris accumulation, animal mortality, damaged agriculture, or disrupted health services; and/or (iii) relevance to human, animal, or plant health preparedness after Hurricane Melissa. The table therefore distinguishes plausible One Health hazards from confirmed post-Melissa morbidity signals; confirmed signals are discussed in the text where data are available.

Effectively tackling this reinforcing loop requires more than the distribution of chlorine tablets and hygiene kits. Addressing acute water insecurity necessitates urgent short-term interventions, including mobile water purification units/systems, water tanker trucks, water and hygiene promotion campaigns, and water sharing within communities [77,78,79]. Understanding human behaviour within crisis situations, especially those related to water security and water use practices, is important in shaping intervention design, risk communication strategies, and operational intelligence [80,81,82]. Prospectively, investment in climate-resilient water purification and distribution systems in Jamaica and CARICOM states with renewable energy-powered redundancies are essential [79,83,84].

### 3.2. Vectors and Vector-Borne Diseases

Vector-borne diseases (VBDs) present a major public health challenge, disproportionately affecting populations in tropical and subtropical regions such as Caribbean SIDS [9,10,11,42,85]. VBDs are primarily transmitted by haematophagous arthropods such as mosquitoes, ticks, and sandflies, resulting in a significant global disease burden which contributes to high morbidity and mortality rates [85,86]. Arbovirus infection risks threaten an estimated 4 billion people worldwide and are expected to increase to 5 billion by 2050 [85,87]. Climate change significantly affects VBDs in humans, influencing temperature, precipitation, humidity, wind speed, and other factors [85,88]. These all affect the reproduction, development, behaviour, and population dynamics of the arthropod vectors of these diseases and the transmission cycles of VBD pathogens [88].

Hurricanes modify the physical and biological landscape in ways that promote rapid vector population growth. Debris can block drains, leaving stagnant water; roofs are lost, leaving people and even homeless persons exposed without shelter, which then prompts increased water storage increasing the risk of larval habitats for *Aedes* and *Culex* mosquitoes; The secondary mechanisms are operational and behavioural; vector-control teams may be displaced or under-resourced, communities may prioritse water storage over container management and damaged homes can increased exposure to mosquito vectors. The effect of Hurricane Irma in 2017 on mosquito abundance led to an eight-fold increase in *Aedes* and *Culex* mosquito species populations in Miami, Florida [89]. An increase in temperature in a warming climate accelerates mosquito development, and pre-crisis analyses for Hurricane Melissa specifically warned that damage to vector control operations and increased standing water would raise mosquito-borne disease risk, including dengue and acute diarrhoeal infections [90]. Additionally, hurricanes may also have a diluting effect on mosquito populations by flushing larvae and eggs out of the environment, causing population crashes in some specialised species, such as *Aedes taeniorhynus*, black salt marsh mosquito, in brackish waters [91].

Jamaica-specific post-Melissa surveillance reports suggest that dengue had not yet translated into a confirmed epidemic signal by late November 2025 [43]. PAHO reported 452 suspected or probable dengue cases in Jamaica in 2025 up to 27 November, with zero laboratory-confirmed dengue cases, two suspected dengue-related deaths that were not laboratory confirmed, and weekly totals remaining below epidemic thresholds since August 2024 [43,92]. By mid-January 2026, the Westmoreland Health Department reported declining mosquito indices, including the Breteau index, following intensified road clearance, fogging, and community vector-control interventions [93]. These data are important and illustrative of how Hurricane Melissa created conditions favourable to arboviral transmission, as available surveillance suggested increased risk potential and local vector proliferation rather than a confirmed dengue outbreak during the reporting period.

**Figure 2:**
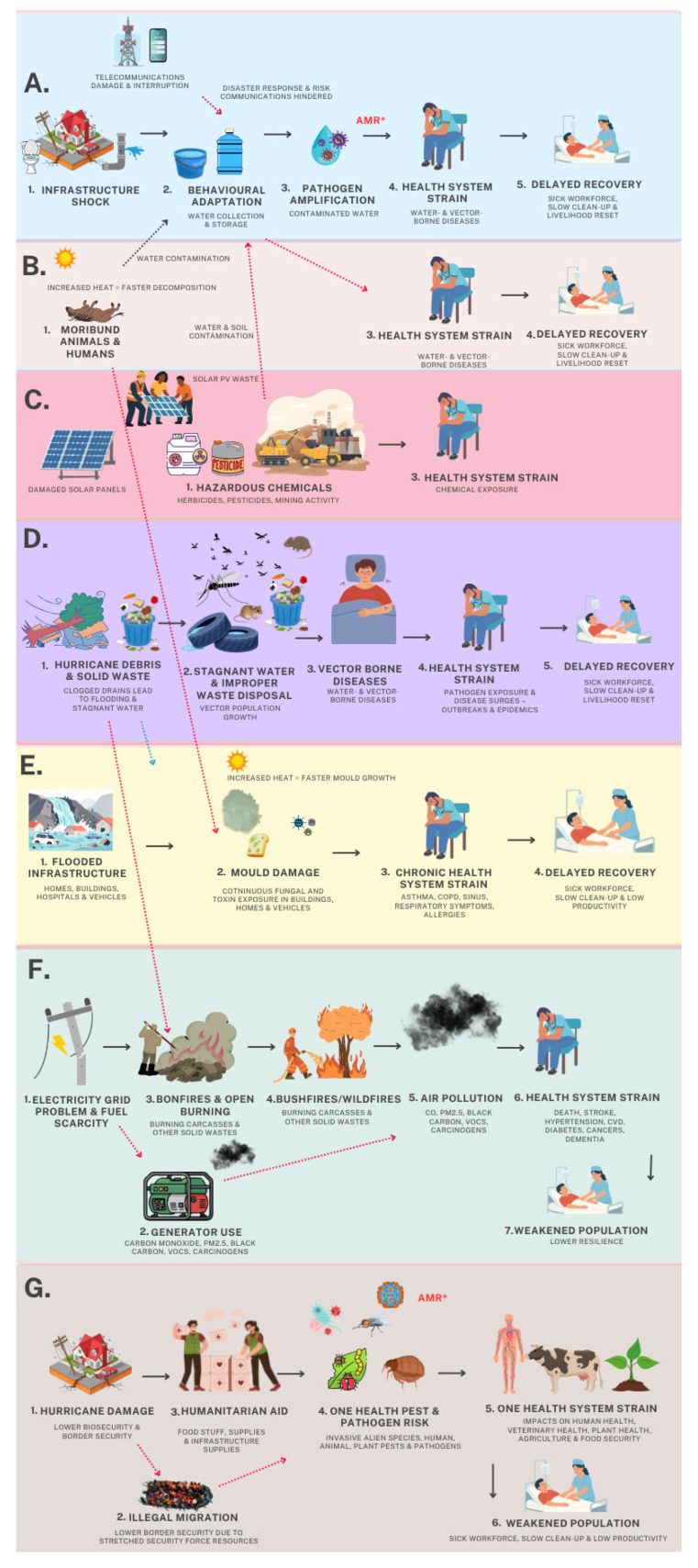
One Health security risk intelligence of the multidimensional impacts of Hurricane Melissa in Jamaica, 2025. “Intelligence” is used here to mean an integrated decision-oriented interpretation of hazard, exposure, vulnerability, surveillance, and response information. The figure was created by the authors as a conceptual synthesis not as a statistical model, using the evidence base and analytic approach described in the text. This conceptual figure illustrates how Hurricane Melissa generated interdependent human, animal, plant, environmental and infrastructural risks across multiple post-disaster pathways. (**Panel A**) depicts the infrastructure–waterborne disease pathway, linking damaged housing, water, sewerage and road systems to unsafe water storage, pathogen amplification, antimicrobial resistance (AMR), health-system strain and delayed recovery. (**Panel B**) shows potential risks arising from moribund animals and human remains, including water and soil contamination, accelerated decomposition under heat, and subsequent health-system burden. (**Panel C**) illustrates potential hazardous chemical exposure pathways associated with damaged solar photovoltaic infrastructure, agrochemicals, pesticides, mining-related materials and other toxic substances. (**Panel D**) depicts debris, stagnant water, poor waste disposal, vector proliferation and vector-borne disease transmission. (**Panel E**) shows flood-damaged infrastructure leading to mould contamination, asthma, allergies, chronic respiratory morbidity and delayed recovery. (**Panel F**) illustrates grid failure, fuel scarcity, generator use, open burning, bushfires or wildfires, air pollution and related cardiopulmonary health-system strain. (**Panel G**) depicts broader potential biosecurity consequences, including reduced border security, illegal migration, humanitarian aid flows, invasive alien species, human, animal and plant pest or pathogen risks, AMR, and weakened population resilience. Numbers within each panel indicate sequential steps in each risk pathway and do not represent quantitative rankings. Solid black arrows indicate primary causal progression within each pathway. Red dashed arrows indicate cross-pathway spillovers or interactions between panels, while blue dashed arrows indicate indirect environmental contamination pathways. The background colours distinguish thematic domains and are illustrative rather than quantitative. The asterisk denotes AMR, antimicrobial resistance, which is treated as a cross-cutting One Health risk amplified by healthcare disruption, contaminated environments, animal losses, inappropriate antimicrobial use, and weakened surveillance.

**Figure 3:**
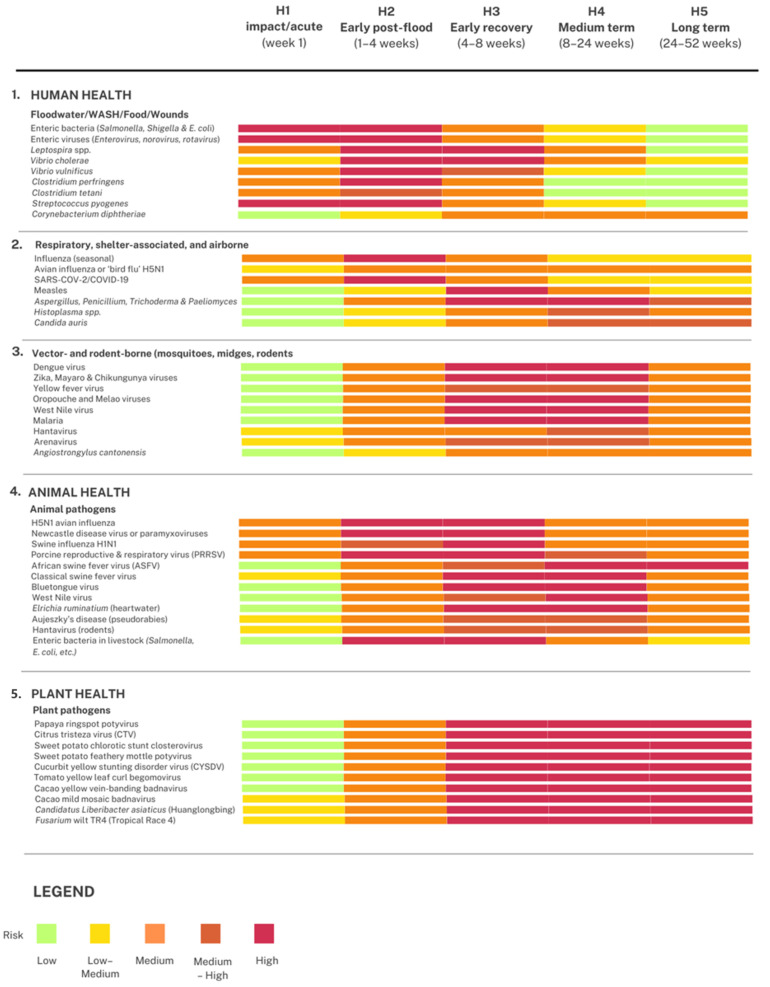
Timeline of diverse One Health infectious disease risks after Hurricane Melissa in Jamaica. **N.B.** The timing and risk scores are qualitative author-derived estimates based on Appendix A, the published literature on post-flood and post-hurricane disease ecology, and Jamaica-specific situation reports. Numbered sections denote major risk domains: 1, human floodwater/WASH/food- and wound-related infections; 2, human respiratory, shelter-associated and airborne infections; 3, human vector- and rodent-borne infections; 4, animal health risks; and 5, plant health risks. H1–H5 indicate post-landfall phases: H1, impact/acute period (week 1); H2, early post-flood period (1–4 weeks); H3, early recovery (4–8 weeks); H4, medium term (8–24 weeks); and H5, long term (24–52 weeks). Colours indicate qualitative relative risk intensity, ranging from low to high, as shown in the legend. The risk estimates are schematic and intended to support preparedness and surveillance prioritisation rather than predict exact outbreak timing.

In a hurricane-modified landscape, a diverse range of vector-borne and rodent-borne infectious diseases and biothreats emerge. The risks associated with each vary regarding the timing of their peak transmission by the mode of transmission and the type of pathogen (Figure 2 and Figure 3 and Table 2). Using a systems-thinking perspective, Figure 2 describes how hurricane-triggered shocks propagate across interconnected human, animal, plant, environmental, infrastructural, and health system domains to generate compound and reinforcing risks in a Small Island Developing State context.

Urbanisation influences the emergence of new vector-borne diseases and the further intensification of others, particularly viral diseases transmitted by *Aedes* spp. mosquitoes [94]. It is the process whereby a large number of people, as part of the proportion of a population living in urban areas, are permanently concentrated in relatively small areas that form a city or settlement [95]. *Aedes* species, such as *Aedes aegypti* and *Aedes albopictus*, often exploit anthropogenically altered aquatic microenvironments such as containers, water storage drums, plant pots, municipal solid waste, discarded tyres, buckets, and certain plant species, e.g., ornamental bromeliads as larval habitats [96]. The Caribbean already experiences endemic and epidemic dengue, with strong associations between rainfall, humidity, waste management, and human cases [8,10,97]. With flooding in the direct aftermath of Hurricane Melissa, the probability of increased dengue and other VBDs transmission in the following 4–8 weeks was high. Other mosquito species with public health relevance are present in Jamaica including the first confirmed report of *Aedes vittatus* [98]. This species presents a public health threat due to its extensive geographic range and proven capacity to transmit yellow fever, dengue, Chikungunya, and Zika viruses [99].

These same ecological conditions support Zika and Chikungunya virus transmission, which have previously swept through the region [100,101]. There have been recent Chikungunya virus outbreaks in the Americas—in Brazil [102], Paraguay [103], Bolivia, and parts of the Caribbean—where the circulation of the ECSA genotype has been identified. Although the Asian genotype continues to circulate at lower levels, its co-circulation with ECSA in the region raises concerns about increased viral adaptation potential and the possibility of recombination or lineage change in the future. Between epidemiological week (EW) 1 and EW 33 of 2025, a total of 212,029 suspected cases of Chikungunya fever were reported, with 124,942 (probable and confirmed), including 245 deaths from chikungunya, with 98% of cases reported in Brazil (*n* = 425,773 cases) [104].

Flood-driven changes in vector habitat and human migration can facilitate its spread into new ecologies. Mayaro virus (MAYV) is a mosquito-transmitted alphavirus, first isolated in Trinidad in 1954, spread throughout the Americas with the majority of MAYV cases being reported from the Amazon basin region, including Brazil, Peru, and Ecuador. With multiple arboviruses in circulation in Caribbean countries poses challenges of coinfection and superinfection necessitating the need to understand the dynamics of multiple infections and resultant virus transmission risks. One study investigating the effects of CHIKV and MAYV coinfection and superinfection on replication dynamics in *Aedes aegypti* found *Ae. aegypti* is capable of transmitting both CHIKV and MAYV concurrently during coinfection however during superinfection, prior infection with either virus significantly reduced the transmission efficiency of the subsequently acquired virus [105]. A study in Trinidad and Tobago was the first to demonstrate the possible co-circulation of Mayaro and Chikungunya viruses and the occurrence of human cases for both diseases during an outbreak in the Caribbean [106]. There is a clear need for more easily accessible diagnostic tools for MAYV infection and additionally a need for ongoing surveillance for the virus, to monitor incidence and spread of MAYV into new areas.

Even if population immunity dampens large-scale epidemics, focal outbreaks can still affect pregnant women, persons with comorbidities, and children. Increasingly, the Caribbean must also consider additional new emergent arboviruses as part of its risk space, such as Oropouche virus (OROV), historically associated with midges and *Culex* mosquitoes in Amazonian and Latin American contexts but now recognised as an emerging concern in the wider Americas [11,104]. OROV originated in Trinidad and Tobago during 1955 and spread across the Amazonian Basin, with recent outbreaks observed in the Caribbean (Cuba, Haiti, and Barbados) and Europe, highlighting the importance of air travel in its dissemination [11,104].

Jamaica has effectively controlled malaria spread by *Anopheles* spp. for decades, but climatic shifts and human mobility mean re-introduction and local transmission remain credible risks, particularly when health systems are distracted by disaster response [107,108]. This may be undermined by the arrival of illegal migrants from Haiti who may be actively infected and transmit the malarial parasite to local competent vector populations [109,110]. Likewise, West Nile virus (WNV), transmitted largely by *Culex quinquefasciatus*, could exploit post-hurricane increases in breeding sites and bird–mosquito contact patterns [61]. These were not the foremost concerns immediately after Hurricane Melissa, but a systems lens requires analysis of a broader hazard portfolio which includes field monitoring of mosquito vectors and heightened clinical surveillance.

Plant health can be negatively impacted by hurricanes specifically through the introduction of biological threats. One study based on an aerobiology transport model (HYSPLIT simulations) supported the notion of Hurricane Ivan causing the introduction of soybean rust (*Phakopsora pachyrhizi*) into the US by moving *Phakopsora pachyrhizi* over the Caribbean from Colombia [111]. Long-distance insect movement caused by extreme phenomena has been previously recorded such as the African locust (*Schistocerca gregaria*) in the Windward Islands (Caribbean) [112,113]. Whiteflies (*Bemisia tabaci*) transported by Hurricane Andrew into Florida from the Caribbean during summer of 1992 was responsible for the introduction of bean golden yellow mosaic virus (BGYMV) and a subsequent epidemic after successive plantings of bean crops [114]. Recent reports of whitefly infestation in neighbouring Cayman Islands prompted the Department of Agriculture Cayman Islands Government to issue an intervention district spraying schedule to control this insect vector [115]. The impact of the passage of Hurricane Melissa on this development may not be immediately identifiable, but it does signal that further research in the Caribbean is urgently needed to understand the impacts of climatic and hydrometeorological events on plant health. Further, the development of climate-plant disease-plant pest predictive models may be advantageous for SIDS to effectively manage emerging risks.

### 3.3. Vector Control

Mass trapping of adult mosquitoes has emerged as a promising complementary strategy for *Aedes* and *Culex* mosquito control, particularly in small-island settings where the spatial scale makes area-wide coverage more feasible. Conceptually, mass trapping entails the deployment of a sufficient density of highly attractive, lethal devices within a defined area such that a meaningful proportion of host-seeking or gravid female mosquitoes is removed from the population on a continuous basis [116]. In contrast to conventional space spraying, which provides transient knockdown, mass trapping aims to exert sustained pressure on vector populations by repeatedly intercepting adult females at critical behavioural stages, most notably when they seek oviposition sites [116]. The deleterious impacts insecticide spraying has on biodiversity such as butterflies and honeybees along with the growing threat of insecticide resistance necessitate an urgent shift away from this vector control strategy [117,118,119,120]. The strongest body of evidence for mass trapping in island and coastal contexts derives from three interconnected technological families: autocidal gravid ovitraps and other lethal ovitraps; passive gravid *Aedes* traps; and powered odour-baited traps, notably the Biogents series (BG-Sentinel, BG-Mosquitaire, and BG-GAT) [121,122]. Odour-baited BG-Sentinel and BG-Mosquitaire traps emulate human scent and convection currents, often augmented by carbon dioxide plumes, to attract host-seeking *Aedes aegypti*, *Aedes albopictus*, and other peridomestic vectors; a fan then draws mosquitoes into a capture bag where they die of desiccation. This eliminates the use of chemical insecticides for vector control with chronic positive benefits to local biodiversity when intensive larval site management (LSM) is paired with these traps to ensure the steady reduction in mosquito population numbers.

On small resort islands in the Maldives, Jahir et al. (2022) implemented an integrated programme combining larval source management with dense deployment of Biogents BG-Mosquitaire CO_2_ traps targeting host-seeking adults and BG-GAT gravid traps targeting ovipositing females [121]. On two islands, the intervention produced near elimination of mosquito populations, while on a third island *Aedes* mosquitoes were absent for more than six months following implementation [121]. Parallel operational documentation from the same resort group indicates that the Biogents trap system enabled a transition away from routine insecticide fogging towards a predominantly insecticide-free management regime, with concomitant reductions in nuisance biting and dengue risk [121].

The ecological objective of post-hurricane vector control should be risk reduction without avoidable harm to biodiversity or public trust. While mass trapping and larval-source management can reduce reliance on broad chemical fogging, any emergency intervention should be monitored for non-target effects, community acceptability, maintenance burden, and disposal of trapped insects and device components. This is especially important in damaged landscapes where pollinators, beneficial insects, and already-stressed ecosystems may be vulnerable to additional disturbance.

On Puerco Island, Palawan, a small island in the Philippines, Knols et al. (2023) implemented a mass-trapping intervention using BG-Mosquitaire CO_2_ traps baited with sugar-fermenting yeast as the carbon dioxide source, in combination with environmental management [123]. The traps, deployed at high density across the island, achieved rapid and sustained suppression of both *Aedes aegypti* and *Culex quinquefasciatus* populations, with near-elimination of these species reported within approximately one year of programme initiation [123].

The default operational response in many jurisdictions has been to intensify space spraying and residual insecticide applications. While these measures can be justified in the acute phase to rapidly suppress adult vectors, their widespread use around shelters and vulnerable populations continue to raise several concerns. This issue was brought into sharp focus in Jamaica where members of a vector control team were attacked physically with stones by upset residents [124]. In humanitarian settings, where trust in authorities may already be fragile, visible and odorous chemical interventions can also provoke anxiety and resistance from affected communities as observed in Jamaica in the direct aftermath of Hurricane Melissa [124]. The exposure of young children, pregnant women, elderly persons, and individuals with chronic respiratory conditions to repeated ULV insecticide fogging in poorly ventilated environments can be problematic [125]. Misuse or overuse of insecticides during emergencies can accelerate insecticide resistance evolution, potentially undermining the efficacy of long-lasting insecticidal nets and indoor residual spraying for malaria and other vector-borne threats [126].

For Jamaica and other Caribbean SIDS facing intensifying hurricane risks under climate change, it is prudent to pre-position mass adult vector elimination trap stocks, include mass trapping modules into national disaster preparedness plans, and integrate with One Health surveillance systems. In such a framework, mass trapping can contribute not only to immediate risk reduction during the aftermath of events like Hurricane Melissa but also contribute to longer-term resilience by reducing dependence on toxic insecticide regimes, preserving insecticide susceptibility, safeguarding pollinator species and food security, and fostering community engagement in sustainable vector management.

### 3.4. Rodent-Borne Pathogens: Hantaviruses, Leptospires, and Arenaviruses

Rodentia is the most abundant and diversified order of living mammals, representing about 43% of the total number of mammalian species [127]. Rodents often inhabit areas in close contact with human populations, their farm animals and or pets. Peri-urban rodents usually represent a nexus between wildlife communities and humans, exposing humans to some zoonoses circulating in these natural ecosystems [127]. In the direct aftermath of a hurricane rodent displacement is triggered by flooding driving them closer to damaged homes and improvised shelters, which can provide them with sufficient shelter and food. Seasonal changes in rodent abundance often do not fully explain seasonal variation in primary human rodent-borne disease cases [128], as longitudinal field studies (>5 years) show a positive but delayed relationship between reservoir abundance and reservoir prevalence [128]. Increased direct and or indirect (faeces, urine or contaminated dust) contact can expose humans to several zoonotic pathogens. Post-hurricane rodent abundance can increase significantly as house mouse (*Mus musculus*) abundance doubled 9 months after two Category 3–5 hurricanes in the US Virgin Islands [129]. Beyond leptospirosis, rodent abundance raises theoretical risk for hantaviruses and arenaviruses (e.g., LCMV), which are transmitted via aerosolised rodent urine and faeces, or direct contact and are endemic in other Caribbean islands [8,42]. While not currently prominent in Jamaican surveillance, a One Health orientation urges vigilance: rodent ecology, agricultural storage practices, and informal settlement patterns can shift rapidly in a post-disaster environment. Eosinophilic meningitis caused by *Angiostrongylus cantonensis* is endemic in Jamaica affecting both adults and children [76,130]. Often cases resolve without sequelae; however, young children are at high risk of neurological damage and death. Thus, vigilance for rodent-borne disease transmission should be practiced and food waste be placed in appropriate waste receptacles out of the reach of rodents in emergency situations.

The health significance of this rodent pathway is immediate and medium term. Leptospirosis risk increases when people contact floodwater, mud, soil or debris contaminated with urine from infected animals, particularly rats. Hantaviruses and arenaviruses are less prominent in Jamaican routine surveillance, but a One Health approach requires vigilance because aerosolised rodent urine and faeces, contaminated dust, agricultural storage, damaged housing and informal settlements can all change exposure intensity [9,97]. Eosinophilic meningitis caused by *Angiostrongylus cantonensis* is endemic in Jamaica and can cause severe neurological disease in children [76,130]. Therefore, rodent surveillance should include trapping indices, species identification, pathogen screening, georeferenced infestation complaints, food-market inspections and linked human syndromic surveillance for febrile, renal, pulmonary and neurological presentations.

Poor solid waste management and informal dumping increase dengue risk in the Caribbean [10]. In Jamaica, nearly five million tonnes of debris and household waste were scattered across western Jamaica, blocking roads, schools and markets [131]. This amplified mosquito and rodent proliferation and concomitant infectious disease transmission. The timing of these risks was staggered: an initial rise in waterborne and rodent-associated infections, followed by a lagged increase in arboviruses as vector populations expanded and human–vector contact intensified (Figure 2).

In response, short-term actions include emergency integrated vector management (IVM), with rapid environmental clean-up of key breeding sites, targeted larviciding, safe adulticiding where appropriate, distribution of bed nets in high-risk settings, and intensive risk communication on safe water storage and container management. Longer-term, Jamaica must move toward structured systematic vector control with reliable solid waste collection, proper drainage and gully management, urban design that minimises stagnant water, and sustained community-based vector surveillance.

### 3.5. Agriculture, Antimicrobial Resistance, and Zoonoses

Antimicrobial resistance (AMR) is a complex systematic challenge inclusive of human, animal, plant, and environmental health. The evolution of bacteria, viruses, parasites, and fungi can result in antimicrobial substances designed as treatments becoming ineffective, threatening lives and livelihoods, increasing the risk of transmission, resulting higher morbidity and mortality rate and the associated costs [132,133]. Agricultural damage after Hurricane Melissa raises two interrelated concerns: classical zoonotic infections and antimicrobial resistance (AMR) dynamics at the human–animal–environment interface. Livestock and poultry in the Caribbean may harbour pathogens such as *Salmonella*, *Campylobacter*, *Listeria monocytogenes*, *Brucella* spp., *Coxiella burnetii*, and shiga toxin-producing *E. coli*, which can enter human populations through contaminated meat, dairy, or environmental pathways (water, soil, dust) when biosecurity and food safety systems are compromised [134].

Mass mortality events among livestock and poultry during disasters tend to generate large volumes of animal carcasses. If these are not rapidly identified, collected, and disposed of safely (e.g., via rendering, deep burial, or controlled incineration), they become foci for bacterial proliferation, attract scavengers and rodents, and can contaminate surface and groundwater. Informal or emergency slaughter of injured animals, often without veterinary inspection or proper offal management, further blurs the boundary between an “animal health problem” and human outbreak. AMR overlays this hurricane-modified landscape. AMR has been identified as a priority threat for the Caribbean by PAHO with support being provided for countries to strengthen surveillance, laboratory capacity, and stewardship across human and animal sectors [135]. The Caribbean Integrated Surveillance System on AMR in Agriculture (CISARA) led by CaribVet, a regional Caribbean animal health organisation, and related initiatives have piloted surveillance of antimicrobial use and resistance in poultry and other sectors.

Several pathways exist in post-disaster situations which may accelerate AMR emergence and dissemination including (1) inappropriate antimicrobial use in humans and animals due to disrupted supply chains, limited clinical stewardship, and increased reliance on international aid due to limited availability, (2) environmental release of AMR pathogens and AMR genes from wastewater and animal waste due to wastewater infrastructural damage, flooded farms, and animal carcasses, (3) reduced AMR surveillance and stewardship as public health resources are constrained and diverted to acute response potentially creating blind spots. Whilst AMR may not produce the immediacy of leptospirosis or dengue, it constitutes a smouldering crisis that can be intensified by the same systemic failures exposed by hurricanes.

Plant health in hurricane struck countries is negatively affected due to wind damage and pathogen and pest dispersal [136]. During the 2004 hurricane season there was widespread citrus canker infections discovered in late 2004 and 2005 and a predictive model was developed to explain storm-related spread of citrus canker [137]. The predictive model revealed that approximately 80% of the hurricane related and subsequent secondary spread of citrus canker would occur over the next 14 months [137].

These impacts of hurricanes on agriculture influence food production, availability and security which negatively impact on nutrition increasing the risks of chronic non-communicable diseases (NCDs) [138]. More focal research on the direct impacts of hurricanes and hydrometeorological events on agriculture is urgently needed in the Caribbean.

### 3.6. Environmental Toxic Chemical Exposure Risks

Strong consideration of infectious disease and toxicological risks to the environment and human health is necessary for preparedness and response strategies to hurricanes in the Caribbean. This recognition is critical as the probability and particular hazards of toxic chemical agents present in floodwaters by chemical spills, biotoxins, solid waste, and sewage can be present. The demonstration of increased chemical pollutants in soils after flooding caused by Hurricanes Sandy and Harvey in the USA [139,140], underscores the need to understand not only biological threats but toxicological threats in the aftermath of hurricanes. Adding diversity to understanding these impacts, one study showed that typhoons can disturb organic contamination in agricultural soils increasing contamination levels of organic chemical pollutants yet also decreasing some inorganic pesticide and polychlorinated biphenyls levels [141]. Consideration of chemicals tracking system to understand the exposure risks in the event of flooding should be urgently pursued as the Chemicals Weapons Convention (CWC) dictates effective governance of hazardous chemicals to which Jamaica is a signatory. Metal mining activity can contribute to chemical contamination of water due to flooding as climate change intensifies [142]. Furthermore, limestone and aggregate mining activity can disrupt geology, ecosystems and water quality.

Renewable energy was identified by Jamaica as a significant source of energy, setting an ambitious aim of generating 50% of its electricity from renewable sources by 2030 committing to sustainable energy and climate resilience [143]. This approach is future thinking, however better design resilience for solar farms is necessary to avoid accumulation of toxic hazardous waste from damaged solar photovoltaic arrays. One megasolar farm, Eight Rivers Solar Park, in Jamaica faced large scale damages from Hurricane Melissa undoubtedly leading to appreciable solar PV waste [144]. Resilient designs for solar farms include removable solar arrays for safe storage ahead of storm and hurricane landfall and these should be widely adopted to reduce financial losses and accumulation of hazardous wastes [145]. The hazardous nature of the waste with leaching potential of Pb above the permissible limits stipulated by various regulatory bodies has been noted [146]. National plans to safely handle hazardous wastes from renewable energy sector are needed in Jamaica and by extension the Caribbean.

The Chemical Weapons Convention (CWC) imposes on Jamaica stringent obligations for the identification, secure storage, monitoring, and traceability of scheduled and relevant industrial chemicals across their life cycle [147]. In the context of Hurricane Melissa, extreme wind and flooding substantially increased the probability of unintentional releases from chemical warehouses, ports, agrochemical depots, laboratories, hospitals, and water-treatment and industrial facilities. Structural failure of tanks, drums, and pipelines may have resulted in dispersion of toxic, corrosive, or environmentally persistent substances into floodwater, soils, sediments, and air, with consequent exposure of humans, livestock, wildlife, and aquatic biota. Robust national CWC-aligned digitised and georeferenced inventories, resilient containment, and post-event environmental surveillance are therefore critical for Jamaica and by extension other Caribbean SIDS.

### 3.7. Invisible Hazards: Mould, Indoor Air Pollution and Open Burning

Due to flooding the built environment can become a reservoir of other health risks. Flooded homes, schools, clinics, and workplaces create ideal conditions for mould and fungal growth, particularly in warm, humid climates [148]. Fungi colonise walls, carpets, insulation, furniture and, critically, ventilation and air-conditioning systems [149]. Post-hurricane mould contamination in hot, humid regions constitutes a persistent environmental health hazard rather than a transient nuisance. After Hurricanes Katrina and Rita, characterisation of air in New Orleans homes revealed markedly elevated concentrations of culturable fungi, (1 → 3,1 → 6)-β-D-glucans, and endotoxin, with predominant genera including *Aspergillus niger*, *Penicillium* spp., *Trichoderma* and *Paecilomyces* [71]. Levels in moderately to heavily water-damaged houses exceeded those previously associated with adverse respiratory outcomes, confirming extensive amplification of fungal and bacterial bioaerosols following prolonged flooding [71]. Immunocompromised populations (e.g., oncology patients) are at high risk in these contaminated environments as witnessed in Houston, Texas after Hurricane Harvey [150].

Bolaños-Rosero et al. (2023) demonstrated persistently elevated indoor fungal loads and diversity one year after the event, with only partial normalisation where comprehensive remediation occurred in Puerto Rico after Hurricane Maria [151]. This highlighted the persistence of exposure risk post-hurricane and flooding events and how monitoring can be used to evaluate risks. These studies indicate that moisture-compromised building envelopes and vehicles in hurricane-affected, warm–humid climates become chronic fungal reservoirs, sustaining increased risks of asthma exacerbation, allergic disease and, invasive mycoses in high-risk hosts [71,150,151]. Awareness and sensitization efforts should be consistent and effective to minimise exposure. They should be inclusive not only to healthcare but also to real estate, construction and architectural professionals. Continuous monitoring of indoor air quality would aid in risk management and proactive interventions as opposed to reactive delayed responses.

Crowded shelters and temporary housing can concentrate respiratory risks: Influenza, COVID-19, RSV, and other respiratory pathogens spread easily where ventilation may be inadequate and people sleep in close quarters [51]. Following Hurricanes Irma and Maria, disaster-related surveillance in five shelters in the US Virgin Islands documented 1130 health-related visits over one month; 17.7% of reasons for visit were acute illnesses, including respiratory syndromes, highlighting that acute respiratory disease constitutes a substantial burden alongside chronic disease management and injury in sheltered populations [152]. The emergence of a variant of H3N2 influenza virus, subtype K, in late 2025 underscored how vaccine mismatch can amplify respiratory burden [153], and this could be exacerbated in densely occupied settings of a post-hurricane environment. Structured, real-time pathogen surveillance and rapid non-pharmaceutical and pharmaceutical interventions within shelters are critical, not optional, components of disaster epidemiology and global respiratory pandemic preparedness.

Simultaneously, damage to the electricity grid, leaving islands without electricity weeks after the storm, prompts widespread use of diesel or gasoline generators, kerosene lamps, and biomass stoves. After Hurricane Maria, the use of diesel generators increased dramatically in Puerto Rico, resulting in the increase in combustion-derived emissions of fine particulate (PM_2.5_), nitrous oxides, carbon monoxide and hazardous air pollutants [154]. The result in increased implications for asthma, COPD, cardiovascular disease, and acute poisoning. Unfortunately, a reported seven deaths due to carbon monoxide poisoning occurred in Jamaica in the aftermath of Hurricane Melissa [155]. The use of low-cost air-quality sensors can assist with monitoring air quality to reduce exposure risks [154]. Open burning of waste can result in wildfires or bush fires as observed in Jamaica in the aftermath of Hurricane Melissa [156], thus guidance on lighting of fires and burning waste in the aftermath of hurricane or storm events are pivotal in reducing bush fire risks. Effective risk communication and public education on the spectrum of air pollution hazards existing after a hurricane recovery should be practiced widely by national and regional emergency and disaster management agencies.

Outdoors in post-hurricane environs, the accumulation of massive volumes of debris and disrupted waste management can lead to open burning of mixed solid waste: plastics, treated wood, electronics, organic matter. Kaya et al. (2025) reported illegal or informal burning of debris in Puerto Rico after Hurricanes Irma and María in 2017 [157]. This open burning practice releases a cocktail of pollutants such as particulate matter, dioxins and furans, polycyclic aromatic hydrocarbons, and heavy metals, exacerbating respiratory and cardiovascular risk, particularly for children, pregnant women, older adults, and people with chronic NCDs [158,159]. The use of sustainable waste management practices for vegetative debris and food waste is preferrable for generation of biogenic methane and biodigestate all input to aid a stable economic recovery and cleaner air post-hurricane impact [160,161].

**Figure 4:**
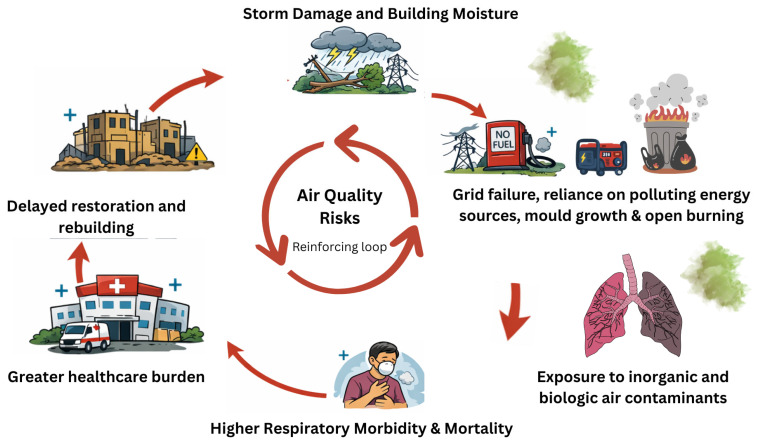
Air-quality exposure risk as a reinforcing loop in the aftermath of a hurricane or hydrometeorological event. Directional (red) arrows indicate hypothesised causal pathways linking storm damage and building moisture to grid failure, reliance on polluting energy sources, mould growth, open burning, exposure to inorganic and biological air contaminants, increased respiratory morbidity and mortality, greater healthcare burden, and delayed restoration and rebuilding. The circular arrows indicate the reinforcing structure of the system, whereby impaired recovery prolongs exposure and further intensifies air-quality-related health risks. The blue “+” symbols denote positive causal polarity, indicating that increases in one system component tend to increase the next component in the loop. Colours are illustrative and do not represent measured quantitative risk levels.

These air-quality hazards illustrate another reinforcing loop: storm damage and building moisture ingress → grid failure and fuel scarcity → reliance on polluting energy sources, mould growth and open burning → higher respiratory morbidity and mortality → increased health system demand just as capacity is constrained → delayed restoration and rebuilding of safer infrastructure (Figure 4).

Clear national guidance against open burning, provision of safe debris collection points, prioritised clearance of waste near health facilities and schools, and targeted support for mould remediation (including PPE and protocols) in critical buildings are urgent measures needed to combat air pollution hazards. Caribbean SIDS must invest in climate-resilient, low-carbon energy systems and integrated waste management that reduce the likelihood that disaster recovery will deepen the air-pollution related healthcare burdens.

### 3.8. Temporary Porosity of Borders, Receipt of Foreign Aid Supplies and Irregular Migration

Hurricanes also reshape social and geopolitical boundaries. Damage to water systems in the neighbouring island of Haiti, was caused by Hurricane Melissa, where an ongoing cholera outbreak has affected 2900 persons since January 2025 [40]. Whilst illegal migration from Haiti to Jamaica is not frequent, the risk of Haitian migrants arriving in Jamaica is not absent, with the arrival of illegal Haitian migrants observed in September 2025 [162]. The risk of the introduction of cholera (*V. cholerae*) to Jamaica should thus be noted particularly during a time when border security resources are very strained due to the impacts of Hurricane Melissa.

During and after Hurricane Melissa, Jamaica’s maritime and aerial assets were heavily engaged in search and rescue, internal logistics, and infrastructure repair, inevitably reducing routine border surveillance [163]. Simultaneously, damage and insecurity in Jamaica and neighbouring countries can stimulate irregular maritime migration, e.g., small vessels moving between Haiti, Cuba, the Dominican Republic, and other islands [162]. From a public health perspective, temporary porosity of borders poses multiple risks. First, the importation or exportation of infectious diseases, e.g., TB, vaccine-preventable diseases (measles, polio, etc.), arboviruses (e.g., Oropouche fever) through mobile, often under-served populations travelling in crowded and unsanitary conditions [164]. Secondly, the illegal movement of animals and animal products (poultry, pigs, fighting cocks, parrots, bushmeat) outside formal sanitary controls, with potential introduction or dissemination of zoonoses and AMR-associated strains can occur [165,166]. Thirdly, there are difficulties in providing care and public health surveillance for irregular migrants, who may avoid health services due to fear of detention or deportation.

The receipt of aid supplies from varied regions and countries carries a risk for the introduction of new pests and pathogens in the acute post-hurricane period as biosecurity, phytosanitary and sanitary protocols are often not as robust as normal. Focus is understandably on providing relief for the population as quickly as possible to alleviate pain, hunger and discomfort. Yet, without rapid emergency protocols to evaluate safety of aid supplies a country can be left most vulnerable to exotic human, plant and animal pathogens inadvertently. Plant health can be negatively affected in a hurricane-modified landscape leading to pest and pathogen accumulation and spread [136]. This impairs food security in the long-term reducing agricultural yields and increasing expenditure to fight plant pests and diseases. More research is necessary to develop rapid screening diagnostics and risk assessments for aid supplies under emergencies and disaster situations.

These dynamics underscore that post-hurricane health security in Jamaica should not be conceptualised solely within national borders; it is intrinsically regional, interconnected with mobility of people, animals, and goods throughout the Caribbean basin. Using the PESTHEEL wheel framework permits diverse risk analysis to understand the spectrum of threats emerging from a post-hurricane response (Table 3) [18].

Table 3 was developed using an adapted PESTHEEL framework to structure the post-Hurricane Melissa One Health risk analysis across eight interdependent domains: political, economic, social, technological, health, environmental, ethical, and legal. The table was not intended as a quantitative risk-ranking tool, but as a structured qualitative synthesis to identify how hurricane-related infectious disease, biosecurity, environmental health, and recovery risks are generated or amplified by wider system conditions. The analysis followed four steps. First, the major post-hurricane One Health threats identified in the preceding sections, including floodwater contamination, vector proliferation, rodent displacement, livestock mortality, antimicrobial resistance, mould exposure, hazardous chemical release, health system disruption, irregular movement of people and animals, and aid-related biosecurity risks, were extracted and grouped thematically. Second, each threat was mapped to the PESTHEEL domain most directly shaping its emergence, amplification, governance, or mitigation. Third, for each domain, the authors identified system “gaps,” defined as structural weaknesses, policy omissions, operational constraints, or institutional vulnerabilities that could increase post-disaster health risk. Fourth, corresponding “opportunities” were formulated as practical governance, surveillance, financing, infrastructure, regulatory, or preparedness actions that could reduce risk and improve future resilience.

This approach allowed the authors to move beyond a pathogen-by-pathogen description and instead examine the systemic conditions through which Hurricane Melissa could generate cascading One Health impacts. For example, vector-borne disease risk was not considered only as an entomological problem, but also as a political issue of emergency coordination, an economic issue of underinvestment in drainage and solid waste management, a social issue of displacement and water storage, a technological issue of surveillance and diagnostics, a health issue of clinical and laboratory preparedness, an environmental issue of altered habitats, an ethical issue of unequal exposure among vulnerable communities, and a legal issue of enforceable post-flood environmental health standards. The table therefore reflects a systems-informed expert synthesis grounded in the reviewed literature, Jamaica-specific post-Melissa observations, where available, and established One Health disaster risk principles.

The PESTHEEL analysis (Table 3) reveals that infectious disease risk is not a narrow “health sector” issue, but a structural property of Jamaica’s political, economic, social, technological, environmental, ethical and legal systems. Addressing these risks after Hurricane Melissa—and before the next major storm—requires deliberate use of these gaps and opportunities to design a truly One Health, systems-informed recovery. This not only benefits Jamaica, but these recommendations have applicability to other SIDS, OCTs, and LMICs impacted by hydrometeorological disasters and emergency events in the face of climate change.

Hurricane Melissa was not just a natural disaster; it was a public health and biosecurity shock that unfolded over weeks, months, and possibly in years to come. Additionally, it exemplifies how climate-charged extreme weather events in the Caribbean create complex, cascading, and often non-linear health risks that demand both systems thinking and a One Health approach.

## 4. Comparative Lessons from Other Hurricane and Flood Settings

The comparative evidence base strengthens, but also bounds, the argument made for Jamaica. North Carolina flood studies after Hurricanes Floyd, Matthew, and Florence showed how faecally contaminated floodwater and flooded soils can increase gastrointestinal and environmental exposure risks [21,44]. Hurricane Katrina demonstrated the speed with which norovirus and acute gastroenteritis can spread in crowded shelters [23]. Hurricane Irma in Miami showed that mosquito abundance can rise sharply after a major storm [89], while the US Virgin Islands experience after Hurricanes Irma and Maria documented both shelter-related acute illness and delayed rodent population shifts [129,152]. Puerto Rico after Hurricane Maria provided evidence for prolonged mould exposure, generator-related air pollution, open burning and the need for low-cost air-quality monitoring after grid failure [151,154,157]. Hurricane Sandy, Hurricane Harvey, and typhoon-related studies showed that chemical and heavy-metal contamination can persist in soils and sediments after flooding [139,140,141]. These studies are not substitutes for Jamaican surveillance data; instead, they define plausible mechanisms, expected time lags, and priority indicators that Jamaica can measure prospectively after Hurricane Melissa and beyond.

## 5. Conclusion: From Disaster to Design

Hurricane Melissa illustrates how an extreme climate-amplified storm can reorganise a country’s health–ecology system within hours. The biothreats Jamaica faces in its aftermath, such as leptospirosis, rotavirus, norovirus, enterovirus, dengue, Zika, Chikungunya, Oropouche, malaria, West Nile, hantavirus, arenavirus, AMR, mould-driven respiratory disease, and indoor and outdoor air pollution, are not random occurrences. They are emergent properties of a complex system where climate change, infrastructure, ecology, governance, and social vulnerability intersect. Framing these threats through systems thinking, PESTHEEL, and One Health moves the focus from individual pathogens to the underlying structures—water and sanitation, agriculture, housing, waste, energy, border governance—that produce vulnerability and resilience. A purely biomedical or purely infrastructural response will fail.

For Jamaica and other Caribbean SIDS, the central challenge is not only to respond to hurricanes like Hurricane Melissa but to redesign systems so that the next hurricane, which climate projections suggest is both inevitable and will likely be more intense, generates fewer and less severe health impacts. This redesign must be locally grounded in national realities and Caribbean experience but also supported by global solidarity and finance commensurate with the scale of climate-driven risk. The question is whether Jamaica and its neighbours can transform this devastating event into a turning point, shifting from reactive recovery to proactive systemic resilience that protects humans, animals and ecosystems together. Achieving this will require sustained political commitment, regional solidarity, and climate finance commensurate with the scale of loss and damage already being experienced. Infectious disease and public health communities, within Jamaica, across the Caribbean, and globally, have a critical role in making the health consequences of hurricanes visible, quantifiable, and central to debates on climate adaptation, disaster risk reduction, and development policy. Focused research on these events only aid in building the lexicography of climate experience in Caribbean SIDS that permit resilient constructs necessary to support sustainable development and robust health systems.

## 6. Recommendations

To support future efforts, we make the following major recommendations for Jamaica and other Caribbean SIDS: (a) to establish a formal multi-sector One Health Biosecurity Intelligence unit within national disaster operations to integrate human, animal, plant, environmental, meteorological, border, chemical and infrastructure data into routine post-hurricane risk briefs and decision-making; (b) to employ phase-based One Health surveillance and early warning as disasters evolve, since actions and activities should be based on timings of risks; (c) to strengthen climate resilient WASH, housing, drainage, waste, and energy systems, as this infrastructure is pivotal for effectively reducing post-hurricane infectious and respiratory disease risks; (d) to integrate hospital, public health, vector, rodent, agricultural, AMR, and biosecurity controls into recovery, shifting from a reactionary to an anticipatory focus for amplification risk assessment, vector reduction, carcass management, AMR stewardship, and post-event biosecurity management of humanitarian aid supplies and other high-risk consignments; and (e) to embed equitable border health and regional learning and training at the heart of resilience planning by prioritising vulnerable groups and remote/socially disadvantaged communities.

## Figures and Tables

**Table 2:** The various phase overviews for human, animal and plant health.

Human Health	Phase	Weeks Post-Landfall	Key Environmental Factors	Main Human Risk Clusters
	Impact & acute flooding	Week 1	Floodwater, trauma, shelter crowding, power loss	Flood/WASH-borne and wound infections, acute respiratory clustering
	Early post-flood	Weeks 1–4	Standing water, debris, waste piles, crowding in shelters	Peak diarrhoeal and leptospirosis diseases, early vector population surges, mould growth, respiratory disease spread in shelters
	Early recovery	Weeks 4–8	Partial drainage, ongoing displacement, mounting garbage piles, temporary/makeshift housing	Vector-borne and rodent-borne disease outbreaks, expanded mould burden, healthcare-related fungal and AMR risks
	Medium term	Weeks 8–24	Rebuilding, informal settlements, compromised housing, disrupted chronic disease healthcare	Sustained arboviral cycles, TB treatment interruption, vaccination schedule interruptions, vaccine-preventable disease outbreaks, chronic mould and air-quality problems
	Long term	Weeks 24–52	Persisting structural vulnerabilities, recurring hydrometeorological events	Entrenched respiratory and NCD impacts, TB, AMR, chronic One Health vulnerabilities
**Animal Health**	**Phase**	**Weeks post-landfall**	**State**	**Driver Main Animal Risk Clusters**
	Impact & acute flooding	Week 1	Damage	Trauma, carcasses, stress, mixing, disruption of farm biosecurity
	Early post-flood	Weeks 1–4	Burial, disposal, treatment	Carcass disposal, contaminated feed/water, expanded vector habitat, pig and poultry crowding
	Early recovery	Weeks 4–8	Restocking	Restocking, informal movements, rebuilding, stable vector/rodent proliferation
	Medium term	Weeks 8–24	Animal flock establishment	Changed landscapes, new stocking densities, persistent vector/rodent pressures
	Long term	Weeks 24–5	Chronic build-up	Entrenched endemicity, trade and movement patterns
**Plant Health**	**Phase**	**Weeks post-landfall**	**State**	**Main Plant Risk Clusters**
	Impact & acute flooding	Week 1	Damage	Mechanical damage by wind, water, and debris, loss of canopy and fruits
	Early post-flood	Weeks 1–4	Regrowth/replanting	Insect vector dynamics (aphids, whiteflies, leafhoppers, mealybugs), introduction risk through humanitarian aid
	Early recovery	Weeks 4–8	Regrowth/replanting	Replanting and planting material quality (e.g., imports)
	Medium term	Weeks 8–24	Crop establishment	Soil pathogen spread and soil chemical contamination due to waterlogging
	Long term	Weeks 24–52	Chronic build-up	Chronic soil contamination by heavy metals

Table 2 was developed using the same narrative synthesis and systems-thinking approach as Figure 3. Phase categories reflect approximate timing after landfall and are intended to support preparedness and surveillance prioritisation rather than to predict exact outbreak onset.

**Table 3:** PESTHEEL analysis of One Health threats in Hurricane Melissa response in Jamaica.

Subsystem		Gaps	Opportunities
Political		Disaster governance architecture has limited formal integration of One Health and biosecurity considerations (e.g., emergency operations centres, cabinet briefings). Post-disaster political communications emphasise infrastructure damage and relief, while subclinical and medium-term infectious risks (e.g., leptospirosis, VBD waves 4–8 weeks later, chronic mould exposure) receive limited high-level framing. Weak institutionalised mechanisms for cross-ministerial decision-making on high-risk aid consignments (agriculture, health, trade, environment, security).	Mandate national emergency operations explicitly include One Health infectious disease risk briefs (floodwaters, vectors, rodents, indoor air/mould, hazardous chemical exposure) at cabinet and parliamentary updates. Use Hurricane Melissa as a catalyst to formalise inter-ministerial infectious disease task forces (health–agriculture–environment–infrastructure-local government) for floods and storms, with pre-agreed triggers for specific interventions (e.g., mass mosquito source reduction, rodent control, mould assessments in schools).
Economic		Economic loss estimates focus on physical assets and agricultural output, rarely quantifying health system costs and productivity losses from flood-borne infections (e.g., leptospirosis, diarrhoeal disease), dengue/arboviral surges, rodent-borne infections and mould-related respiratory morbidity. No routine valuation of long-term costs of inadequate mould remediation (in homes, schools, workplaces, vehicles) or of recurrent dengue and leptospirosis outbreaks exacerbated by underinvestment in drainage and vector control.	Integrate health-impact and productivity modelling (including DALYs and economic costs) of floodwater-borne, vector-borne and mould-related diseases into post-Hurricane Melissa reconstruction and climate-finance proposals. Justify and secure investment in drainage, solid-waste management, urban design and housing standards as cost-effective infectious disease prevention measures, not merely engineering projects. Include preventive health and One Health infrastructure (vector control management, rodent control, mould remediation capacity) as line items in macroeconomic recovery plans and negotiations with IFIs.
			Climate and health financing architecture that targets cascading One Health challenges intrinsically linked to hurricanes and other hydrometeorological events (e.g., national parametric insurance products).
Social		Public risk communication is often inconsistent, delayed or overly technical messaging on floodwater infectious risks (e.g., leptospirosis, faecal contamination), rodent exposure, and mould in homes and shelters. Cultural practices (e.g., children playing in flood waters, informal waste dumping, water storage in open containers) persist without behaviourally informed interventions, amplifying exposure to pathogens. Crowded shelters and informal post-storm living arrangements increase respiratory disease risk, but community understanding of ventilation, masking, and cough etiquette is variable.	Co-design targeted, plain-language messages on leptospirosis, diarrhoeal disease, arboviruses, rodent exposures, and mould risks and make risk messaging understandable, tangible, and actionable. Mobilise local and regional celebrities, community and faith-based organisations to lead neighbourhood clean-up and mosquito source reduction campaigns, combined with advice on rodent control and safe post-flood behaviour. Prepare and record messaging ahead of disaster events ready to be deployed as soon as communications are reactivated (proactive vs. reactive). Develop community messaging and toolkits for post-flood home/vehicle checks (stagnant water, rodent signs, visible mould, ventilation), enabling people to act as first-line risk detectors.
Technological		Limited deployment of environmental sensing and decision-support tools (e.g., real-time rainfall–flood modelling and vector borne disease EWS) in routine disaster operations. Lack of digitised hazardous chemical management system to enable georeferencing and tracking of hazardous chemicals and modelling of exposure risks. Lack of wastewater surveillance to quantify pathogen risks and forecast infectious outbreaks and epidemics.	Develop and implement integrated climate–hydrology–health-mobility early warning models to forecast clusters of infectious disease cases based on rainfall, flooding, and vector indices. Develop digitised hazardous chemical management system to enable georeferencing and tracking of hazardous chemicals and model exposure risks. Issue standard technical guidelines for mould risk assessment (moisture mapping, sampling strategies, HVAC inspection), wastewater surveillance and low-cost mould remediation methods appropriate for tropical, resource-limited contexts.
Health	Human	Lack of clinical awareness of the spectrum of biothreats in a nuanced hurricane-modified environment. Mould-related respiratory disease and exacerbation of asthma, COPD, and allergenic conditions are often under-recognised and under-coded in surveillance systems post-event.	Embedding biosecurity and health security threats in specific disaster and emergency response environments for professional training (medicine, veterinary, public). Implement standardised, syndromic respiratory and allergy surveillance, explicitly attributing possible mould-related exacerbations to inform remediation priorities.
	Animal	Coordination between human health and veterinary/public health services for vector/rodent surveillance and control remains fragmented.	Create joint human–animal–vector surveillance teams to monitor wastewater, rodents, livestock, companion animals and vectors in high-risk areas, feeding into health alerts and interventions.
	Plant	Lack of rapid diagnostic testing for plant pathogens and plant pests on emergency/disaster aid supply consignments.	Expand plant-health surveillance and risk assessment for introduced or redistributed plant pests and pathogens, which indirectly affect human nutrition, pesticide use and livelihoods.
Environment		Post-Hurricane Melissa environmental assessments with limited structured sampling of floodwaters, soils and sediments for pathogenic microorganisms, chemical pollution, rodent activity markers, and mould reservoirs. Lack of mould remediation guidelines and open burning guidelines under emergency and disaster situations.	Incorporate microbiological and rodent-ecology components into post-flood environmental surveys, including testing of standing water and high-contact sediments. Prioritise investment in “healthy infrastructure”: climate-resilient drainage, covered drains, regular gully maintenance, and integrated waste systems that reduce mosquito breeding and rodent refuges. Innovate temporary housing solutions for displaced persons due to disasters and emergencies with adequate WASH and biosecurity integrity.
Ethics		Vulnerable groups (children, older adults, persons with chronic lung disease) are often least able to move out of mould-infested or vector- and rodent-prone housing and exposed to fogging. Households in remote rural communities, informal settlements, hillside or floodplain locations, and small offshore or coastal communities can experience delayed or reduced access to search-and-rescue, medical care, clean water, and reconstruction support relative to more visible urban areas. Low-income groups, renters, informal workers, and persons without secure land tenure are more likely to occupy structurally weak, flood- and mould-prone housing, and to reside in mosquito- and rodent-dense environments, yet they have the least capacity to relocate, remediate, or retrofit their dwellings. Decisions that tolerate long-term mould contamination, recurrent flooding, or chronic vector infestation for the sake of rapid short-term recovery effectively shift risk and cost onto children and future generations, especially in already disadvantaged communities.	Develop transparent, publicly discussed criteria for prioritising shelters, communities and facilities for early WASH restoration, vector/rodent control, and mould remediation, with specific weighting for populations at heightened vulnerability. Incorporate explicit equity and human-rights benchmarks into national disaster plans (e.g., minimum time-to-rescue and time-to-aid targets for remote and high-risk communities). Establish mechanisms for community feedback, grievance and independent review of disaster decisions that appear to produce or perpetuate inequities in rescue, aid, or environmental remediation, and use these findings to refine ethical guidelines before subsequent events. Embed the principle that recovery investments should reduce, not entrench, structural vulnerability, for example, by prioritising resilient housing, drainage, and indoor environmental quality in low-income communities.
Legal		Disaster and public health laws may not include explicit triggers or thresholds for mandatory vector-control, rodent-control, or mould-remediation actions in public facilities post-flood. Absence of enforceable indoor environmental health standards (e.g., moisture and mould criteria) limits the legal leverage to insist on remediation in rental properties, workplaces or public buildings. Limited explicit legal provisions for rapid environmental disease-risk assessments (including microbial, vector and mould assessments) as a standard component of post-disaster obligations.	Amend relevant public health and housing legislation to define minimum standards and enforcement mechanisms for post-flood mould and vector control in public and rental properties. Include vector, rodent, chemical, and mould risk-management clauses in building codes and environmental regulations, especially for critical infrastructure (schools, hospitals, shelters). Codify requirements for integrated post-disaster environmental and infectious disease assessments, with mandated reporting and accountability, ensuring that floodwater pathogens, vectors, rodents, chemical levels, and mould all fall within the legal remit.

PESTHEEL refers to political, economic, social, technological, health, environmental, ethical, and legal domains. Table 2 was constructed by identifying major post-Hurricane Melissa One Health threats discussed in the manuscript, mapping each threat to the PESTHEEL domain that most directly influences its emergence or control, identifying domain-specific system gaps, and formulating corresponding opportunities for recovery, preparedness, and resilience. The table represents a structured qualitative synthesis rather than a statistical risk assessment.

## Data Availability

No new data were created or analysed in this study.

## Appendix A

Appendix A provides a qualitative phase-based mapping of selected human pathogens and pathogen groups that may become epidemiologically important after Hurricane Melissa. The table is intended to indicate the relative likelihood, public health salience, and operational priority of each pathogen across five post-landfall periods: Week 1, 1–4 weeks, 4–8 weeks, 8–24 weeks, and 24–52 weeks. The ratings do not represent measured incidence, prevalence, case counts, or quantitative risk estimates for Jamaica. Instead, they represent an expert-informed synthesis based on known post-flood disease ecology, pathogen incubation periods, vector and rodent population dynamics, WASH disruption, shelter crowding, health system strain, seasonality, and the peer-reviewed and grey literature cited in the manuscript.

The abbreviations used in the following tables are defined as follows: H = high relative concern or priority; M = moderate relative concern or priority; L = low relative concern or priority; L–M = low to moderate relative concern; and M–H = moderate to high relative concern. These categories should be read comparatively within each pathogen group and time phase rather than as absolute probabilities. A rating of H indicates that conditions in that phase are highly favourable for exposure, amplification, detection, or clinical burden, or that the pathogen requires heightened surveillance and preparedness. A rating of M indicates plausible but more context-dependent risk, requiring routine surveillance and targeted response capacity. A rating of L indicates lower expected risk during that period, while L–M and M–H indicate transitional or conditional risks depending on local ecological, epidemiological, immunological, infrastructural, or behavioural conditions.

**HUMAN HEALTH—WEEK-BY-WEEK PHASED TIMELINE**

**A. Phase overview**
  **Phase**	**Weeks Post-Landfall**	**Key Environmental Drivers**	**Main Human Risk Clusters (Examples)**
  1. Impact & acute flooding	**Week 1**	Floodwaters, trauma, shelter crowding, and power loss	Flood/WASH-borne and wound infections; acute respiratory clustering
  2. Early post-flood	**Weeks 1–4**	Standing water, debris, waste piles, and crowding in shelters	Peak diarrhoeal + leptospirosis; early vector and rodent population amplification; intense mould growth; respiratory spread in shelters
  3. Early recovery	**Weeks 4–8**	Partial drainage, ongoing displacement, rubbish piles, and temporary housing	Vector-borne outbreaks; rodent-related risks; expanding mould burden; healthcare-associated fungal/AMR risks
  4. Medium term	**Weeks 8–24**	Rebuilding, informal settlements, compromised housing, and disrupted chronic care	Sustained arboviral cycles; TB treatment interruption; vaccine-preventable outbreaks; chronic mould and air-quality problems
  5. Long term	**Weeks 24–52**	Persisting structural vulnerabilities and recurring climate extremes	Entrenched respiratory and NCD impacts; TB; AMR; chronic One Health vulnerabilities
**B. Human pathogens mapped to phases**

1. **Floodwater/WASH/food- and wound-borne**
  **Pathogen/Group**	**Week 1**	**1–4 Weeks**	**4–8 Weeks**	**8–24 Weeks**	**24–52 Weeks**	**Notes (Drivers)**
  **Enteric bacteria** (*Salmonella*, *Shigella*, *E. coli*, *Campylobacter*, *Clostridium difficile*)	H	H	M	L–M	L	Pit latrine overflow; sewage contamination of water; unsafe food preparation; disrupted refrigeration; compromised handwashing and hygiene
  **Enteric viruses**—enterovirus, norovirus, and rotavirus [17]	H	H	M	L–M	L	Highly infectious; spread in shelters, schools, and crowded homes; water and person-to-person
  *Leptospira* spp. [33]	M	H	H	M	L–M (rainy periods)	Rodent urine in floodwaters; highest when people wade/work in water during clean-up (1–6 weeks)
  *Vibrio cholerae* [37]	L–M (context dependent)	H (if introduced)	H	M	L–M	Saline/brackish floodwater + WASH breakdown; explosive outbreaks if seeded
  *Vibrio vulnificus* [38]	M	M–H	M	L–M	L	Brackish/coastal flooding; wound and seafood exposure
  *Clostridium perfringens* [14]	M	H	M	L	L	Inadequately cooked/stored food; bulk feeding; power loss
  *Clostridium tetani* [39]	M	M–H (in under-immunised)	M	L	L	Wounds from debris, metal, and soil; risk shaped by tetanus coverage
  *Streptococcus pyogenes* [40]	H	H	M	L–M	L	Skin/soft tissue infections in wounds; additional pharyngitis in crowded shelters
  *Corynebacterium diphtheriae* [41]	L	L–M	M	H (with immunity gaps)	M	Outbreak risk in low-coverage pockets; person-to-person in shelters and schools
  *Angiostrongylus cantonensis* [47]	L	L–M	M	M	M	Related to rat–gastropod cycles and food hygiene; may emerge when foraging/crop patterns change
2. **Respiratory, shelter-associated, and airborne**
  **Pathogen/Group**	**Week 1**	**1–4 Weeks**	**4–8 Weeks**	**8–24 Weeks**	**24–52 Weeks**	**Notes**
  Influenza (seasonal)	M	H (crowded shelters)	M	L–M (seasonal)	L–M	Indoor crowding; poor ventilation
  H5N1 “bird flu” [15]	L–M	M	M	M	M	Risk if infected birds present; exposure during carcass disposal, live birds, and markets
  COVID-19	M	H	M	L–M	L–M	Aerosol transmission in shelters, camps, and clinics; may surge with mixing
  Measles [19]	L	L–M	M	H (if immunity gaps)	M	Highly transmissible; appears weeks–months later if coverage poor
  *Mycobacterium tuberculosis* [36]	L–M	L–M	M	H	H	Treatment interruptions, overcrowding, and under-nutrition; medium–long term
  *Aspergillus*, *Penicillium*, *Trichoderma*, and *Paecilomyces* [42]	L	M	H	H	M–H	Mould proliferation in damp buildings; immunosuppressed at greatest risk
  *Histoplasma* [44]	L	L–M	M	M–H	M	Disturbed bat/bird droppings during demolition/clean-up
  *Candida auris* [45]	L	L–M	M	M–H	M–H	Healthcare associated; emerges with ICU crowding, broad-spectrum antibiotics, and poor IPC
3. **Vector- and rodent-borne (mosquitoes, midges, rodents)**

Most vector-borne risks lag flooding by at least **1–3 weeks** (mosquito life cycle; vector habitat formation), often peak during **weeks 4–12**, then persist in susceptible ecologies.

**Pathogen**	**Week 1**	**1–4 Weeks**	**4–8 Weeks**	**8–24 Weeks**	**24–52 Weeks**	**Notes**
Dengue virus [21]	L	M	H	H	M	*Aedes* breeding surge in containers, debris, and blocked drains
Zika virus [24], Mayaro virus [23], and Chikungunya virus [28]	L	M	H	H	M	Similar to *Aedes*/daytime-biting dynamics; transplacental risks for Zika
Yellow fever virus [26]	L	M	M–H (if present)	M–H	M	Depends on local ecology/immunisation; sylvatic and urban cycles
Oropouche & Melao viruses [29]	L	M	H	H	M	*Culicoides* midges/mosquitoes; thrive in damp organic-rich habitats
West Nile virus [30]	L	M	H	H	M	*Culex* mosquitoes; bird–mosquito cycles; wetlands and standing water
Malaria [46]	L	M	H (if vectors present)	H	M	*Anopheles* breeding in new water bodies; depends on local endemicity/importation
Hantavirus [18]	L–M	M	M–H	M–H	M	Rodent displacement, causes nesting in new structures; aerosolised excreta
Arenavirus [18]	L–M	M	M	M–H	M	Rodent hosts; more structural medium-term risk
*Angiostrongylus cantonensis* [47]	L	L–M	M	M	M	Related to rat–gastropod cycles and food hygiene; may emerge when foraging/crop patterns change

**C. Animal pathogens**

1. **Phases (animals)**
  **Phase**	**Weeks**	**Drivers**
  A1. Impact & acute	1	Trauma, carcasses, stress, mixing, and disruption of biosecurity
  A2. Early post-flood	1–4	Carcass disposal, contaminated feed/water, vector habitat, and pig and poultry crowding
  A3. Early recovery	4–8	Restocking, informal movements, and rebuilding; stable vector amplification
  A4. Medium term	8–24	Changed landscapes, new stocking densities, and ongoing vector/rodent pressures
  A5. Long term	24–52	Entrenched endemicity; trade and movement patterns
2. **Animal Pathogens**
  **Pathogen/Group**	**Week 1**	**1–4 Weeks**	**4–8 Weeks**	**8–24 Weeks**	**24–52 Weeks**	**Notes**
  H5N1 avian influenza [15,16]	M	H	H	M	M	Stress, mixing, and wild bird contact; carcass disposal and wet markets
  Newcastle disease virus (NDV)/paramyxovirus [16]	M	H	H	M	M	Similar drivers as H5N1; lapses in vaccination/biosecurity
  Swine influenza [28]	M	M–H	H	M	M	Pig crowding, mixing, and transport; aerosols in barns
  Porcine reproductive and respiratory syndrome virus (PRRSV) [28]	M	H	H	M–H	M	Highly biosecurity sensitive; stress and stocking changes
  African swine fever [23]	L	M	M–H (if introduced)	H	H	Introduced via pigs/pork; persists in environment and products
  Classical swine fever [26]	L–M	M	H	H	M	Similar to ASF but vaccine preventable where programmes exist
  Bluetongue virus [21]	L	M	H	H	M	*Culicoides* vectors; wetlands and damp soil; affects ruminants
  *Ehrlichia ruminantium* (heartwater) [34]	L	M	M–H	H	M	Tick-borne; altered grazing and wildlife–livestock interface
  West Nile virus [30]	L	M	H	H	M	Equids and birds; vector dynamics as in humans
  Aujeszky’s disease (pseudorabies) [26]	L–M	M	M–H	M–H	M	Swine stress, movements, and inter-farm contacts
  Hantavirus (rodent) [19]	L–M	M	M–H	M–H	M	Rodent population shifts; flood-related rodent displacement
  Enteric bacteria in livestock (*Salmonella*, *E. coli*, etc.)	M	H	H	M	L–M	Contaminated feed/water, stress, manure handling, and slaughter disruptions

**D. PLANT HEALTH TIMELINE**

For plants, post-hurricane risk is driven by:

- Mechanical damage and loss of canopy.
- Vector dynamics (aphids, whiteflies, leafhoppers, mealybugs).
- Replanting and planting material quality.
- Soil pathogen spread and altered waterlogging.

Plant pathogens

Most of these viruses are vector-borne (whiteflies, aphids, etc.) and will peak once vector populations rebound and new susceptible growth is available.

**Pathogen/Group**	**0–2 Weeks**	**2–8 Weeks**	**8–24 Weeks**	**24–52 Weeks**	**Notes**
Papaya ringspot potyvirus [17]	L	M	H	H	Aphid-transmitted; new papaya plantings and flush growth vulnerable
Citrus tristeza virus (CTV) [17]	L	M	H	H	Aphid-vectored; damaged citrus groves stressed and re-grafted
Sweet potato chlorotic stunt closterovirus [17]	L	M	H	H	Whitefly-borne; spread in replanted vines
Sweet potato feathery mottle potyvirus [17]	L	M	H	H	Seed/vegetative material and vector contribution
Cucurbit yellow stunting disorder virus (CYSDV) [17]	L	M	H	H	Whitefly-transmitted; cucurbit replanting after storm
Tomato yellow leaf curl begomovirus [17]	L	M	H	H	Whiteflies in tomato production; major threat after re-establishment
Cacao yellow vein-banding badnavirus [17]	L–M	M	H	H	Chronic; storm damage may change vector/host interface
Cacao mild mosaic badnavirus [17]	L–M	M	H	H	As above; long-term canopy recovery phase
*Candidatus Liberibacter asiaticus* (Huanglongbing/citrus greening) [35]	L–M	M	H	H	Psyllid-vectored; long-term chronic; storm may accelerate spread via movement of planting material and grafting
*Fusarium* wilt TR4 (Tropical Race 4) [43]	L–M	M	H	H	Soil-borne; spread via floodwaters, contaminated tools and planting material; becomes apparent over months–years
**Footnote:** H = high relative concern or operational priority; M = moderate relative concern or operational priority; L = low relative concern or operational priority; L–M = low to moderate relative concern; M–H = moderate to high relative concern. Ratings are qualitative and phase-specific. They reflect expected post-hurricane conditions, pathogen ecology, exposure pathways, incubation periods, vector/rodent dynamics, shelter crowding, WASH disruption, and health system vulnerability. They are not measured as Jamaica-specific incidence rates or statistical risk estimates.					
